# Moyamoya Disease: Clinical and Radiological Characteristics in Adult Greek Patients

**DOI:** 10.3390/jcm12185951

**Published:** 2023-09-13

**Authors:** Sofia Vassilopoulou, Argyro Tountopoulou, Eleni Korompoki, Georgios Papageorgiou, Dimitrios Kasselimis, Georgios Velonakis, Achilles Chatziioannou, Constantin Potagas, Konstantinos Spengos

**Affiliations:** 1 Stroke Unit, 1st Department of Neurology, Eginition Hospital, National and Kapodistrian University of Athens, 11528 Athens, Greece; 2 Department of Clinical Therapeutics, National and Kapodistrian University of Athens, 11528 Athens, Greece; 3 Neuropsychology and Language Disorders Unit, Eginition Hospital, National and Kapodistrian University of Athens, 11528 Athens, Greecedkasselimis@gmail.com (D.K.);; 4 Department of Psychology, Panteion University of Social and Political Sciences, 17671 Athens, Greece; 5 2nd Department of Radiology, National and Kapodistrian University of Athens, 12462 Athens, Greece; 6 1st Department of Radiology, National and Kapodistrian University of Athens, 11528 Athens, Greece; 7 Department of Neurology, Hygeia Hospital, 15123 Athens, Greece

**Keywords:** Moyamoya, stroke, neuroimaging, demographic, neurocognitive

## Abstract

Background and purpose: The aim of our study is to present, for the first time, the clinical, radiological, and neurocognitive characteristics of Greek adult patients with Moyamoya disease (MMD). Methods: We analyzed prospectively collected data of 12 patients referred to our department from 2004 to 2019. All patients underwent a thorough diagnostic work up, including extensive clinical, neuroradiological, and neurocognitive assessment. Results: Our study population consisted of 7 females and the median age at the time of the diagnosis was 43.5 years. No patient had a positive family history of the disease and roughly 50% were hypertensives. Ten patients presented with transient or permanent cerebrovascular ischemia and two patients suffered from hemorrhagic complications. The median NIHSS was 7.5 (0–23) and clinical status remained stable during follow-up with conservative treatment in most of the patients. The majority (83.3%) had bilateral disease confirmed by DSA. All lesions exclusively affected the anterior circulation, with 50% of patients presenting with stenoocclusive changes. No aneurysm or AVM were revealed. The most common neurocognitive deficits were in the executive and language domains. Conclusions: Our MMD patients had a later onset of the disease and an absence of familial occurrence. The most common manifestation was ischemia, transient or permanent, and all lesions affected the anterior circulation, whereas no vascular malformations (AVM, aneurysms) were demonstrated in brain imaging. These findings in Greek patients imply a probable different, Mediterranean phenotype.

## 1. Introduction

Moyamoya is a rare, chronic cerebrovascular disorder. It is characterized by progressive stenotic and occlusive lesions of the terminal internal carotid artery (ICA) and intracranial arteries, which leads to the development of a characteristic abnormal, fragile vascular network at the base of the brain. This characteristic network resembles a “puff of smoke”, from which the name of this disease is derived (Moyamoya in Japanese language) [1].

The etiology and pathogenesis of Moyamoya Angiopathy (MMA) are largely unknown. In Moyamoya disease (MMD), there is no underlying cause and thus it is characterized as idiopathic. The angiopathy affects both children and adults and can present with cerebral ischemia and infarction or with hemorrhagic lesions. Cognitive deficits, migraine-like headaches, seizures, and various movement disorders, most commonly irregular jerks, stiffness, and muscle cramps, are also symptoms of the disease [2,3,4,5]. The absence of any symptoms, although rare, has also been reported both in Asian and Caucasian populations [6,7], although completely asymptomatic MMA may be questionable [8].

MMD occurs all over the world, but the frequency in Western countries is notably low compared with Asia. In East-Asian countries, including East-India, China, Taiwan, and particularly in Japan, the MMA incidence rate is reported up to 0.54 per 100,000, whereas MMA in Western countries is reported to be 10 times less frequent (from 0.047 to 0.086 per 100,000) [9,10,11,12,13,14,15]. However, the frequency of MMA is probably underestimated outside East-Asia, as shown by the delay of its diagnosis in European populations [16]. Despite limited data, some differences among ethnicities have already been recorded, such as the disease’s first manifestation, familial occurrence [17], age of the appearance, female predominance, and radiological characteristics.

Limited data exist for Caucasian Moyamoya patients and even less exist for patients in Mediterranean area [18,19,20]. The aim of this study is to present the demographic, clinical, and radiological characteristics of a series of Greek adult patients with MMD, aiming to enrich the sparse information for this peculiar disease.

## 2. Methods

### 2.1. Study Population

This is a prospective study of adult patients >18 years old, with angiographically confirmed Moyamoya angiopathy who were hospitalized in the Stroke Unit or examined in the outpatient Stroke Clinic of Eginition University Hospital, Athens, Greece, from 2004–2019, which is a referral center for a variety of rare neurological diseases.

**Figure 1:**
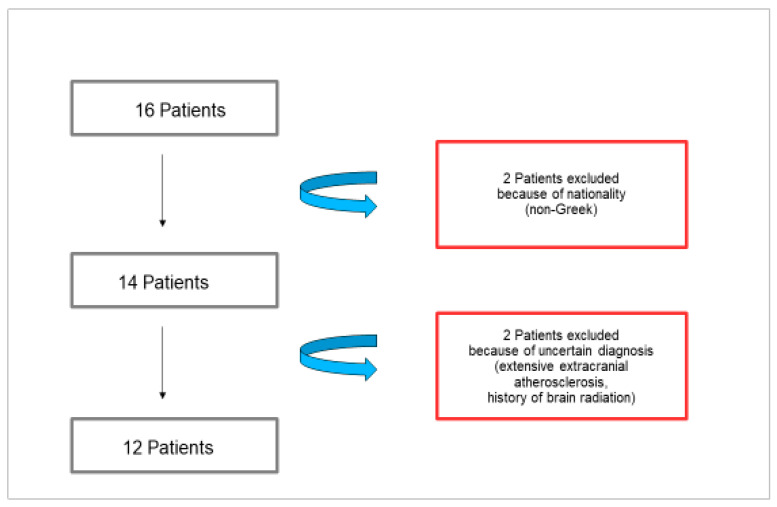
Schematic illustration of our patients’ exclusion criteria.

Sixteen Μoyamoya Angiopathy (MMA) patients were identified. The patients whose angiopathy had been proven to be from another cause (e.g., atherosclerosis or brain radiation lesions) were excluded, so were the patients with a nationality other than Greek. We finally evaluated 12 patients with MMD (Figure 1).

### 2.2. Demographic Data

Patients’ sex, age, laterality, and the presenting symptom (transient ischemic attack (TIA), ischemic stroke, and intracranial hemorrhage (ICH)) of the angiopathy were recorded. Hypertension, dyslipidemia, and smoking were recorded as stroke risk factors, whereas other associated and non-associated diseases or comorbidities such as headache and asthma were also recorded. Extensive familial history was recorded and family members were also examined accordingly. Stroke severity was assessed using the National Institute of Health Stroke Scale (NIHSS) and the outcome was assessed using the modified Rankin Scale (mRS). The medical or surgical treatment at each patient’s discharge was also documented.

### 2.3. Diagnostic Work up

The diagnostic work up included blood investigations (complete blood count, erythrocyte sedimentation rate (ESR), C-reactive protein, vasculitis profile, thyroid profile, and anti-phospholipid antibody profile), cardiac diagnostic work-up (including electrocardiogram, chest X-ray, transthoracic, and, if indicated, transesophageal heart ultrasound), magnetic resonance imaging (MRI), angiographic work-up (extra- and intracranial color-coded ultrasonography of vascular network, magnetic resonance angiography (MRA) and/or digital subtraction angiography (DSA)), and, in most cases, CSF examination. An additional work-up was done as indicated on an individual basis.

### 2.4. Follow up Data

In most patients, the follow up data were obtained from the last clinical visit. In those cases where this was not feasible, we tried to obtain data from a telephone interview either with the patient or with their treating physician.

### 2.5. Neurocognitive Assessment

Neurocognitive status was assessed based on five main cognitive domains: language, episodic memory, executive functions/behavioral deficits, working memory/attention, and visuospatial processing (details on the instruments used has been described elsewhere [21]). Patients were examined either during their hospitalization or at the follow up visit.

### 2.6. Imaging Studies

MRI−MRA were performed both at the time of diagnosis and at a later time during patients’ follow up visit (3T Achieva TX Philips MRI scanner/Philips, Best, The Netherlands). The ischemic lesions and their underlying vascular territory were divided into ACA, proximal stem, distal stem/superior- inferior division of MCA, PCA territory, and cortical branch infarcts. DSA was performed (Allura Xper FD20, Tech ID 023000201, Philips, Amsterdam, The Netherlands) or evaluated by an interventional radiologist. Suzuki classification was based on the most affected side in those patients with bilateral vessel involvement [22].

### 2.7. Statistical Analysis

For continuous variables, the mean, standard deviation and range or median, 25th and 75th percentiles, and range were used after testing for normal distribution. The Shapiro–Wilk test for normality was applied. For categorical variables, the frequencies and percentages were used. All statistical analyses were performed using Stata/IC version 15.1 (StataCorp, 4905 Lakeway Drive, College Station, TX, USA).

## 3. Results

### 3.1. Demographic Characteristics

We evaluated 12 patients—7 women and 5 men (ratio of 1.4/1, respectively). The median age at the time of diagnosis, was 43.5 years (Table 1). No patient had a family history of Moyamoya or of any other angiopathy; this was reported by the patients themselves and by their family members (in all but Patient 10) as family members’ brain images were not available for any of our patients. Ten patients had bilateral involvement and only two had unilateral disease (83% vs. 16.7%, respectively).

The patients’ most common first clinical manifestation was TIA (58.34%). In five patients, a subsequent ischemic stroke (41.67%) was recorded. Three patients (25%) were admitted to the hospital with an ischemic stroke—without any previous symptoms. Only two patients (16.67%) on our registry had a hemorrhagic manifestation as the first symptom. One patient experienced a deep ICH and another had a subdural hematoma, for which he was hospitalized and the diagnostic work up revealed the presence of MMD. The median NIHSS score at diagnosis was 7.5 points.

Ten of our patients had at least one cerebrovascular risk factor (83.3%). The most common was hypertension, but none of the patients were taking antihypertensive medication. In our population, we recorded associated and various non-associated-diseases. Two patients complained of headache, two women had congenital eye-problems (severe in Patient 1), and another two women had a history of an autoimmune disease (Ankylosing spondylitis in Patient 3 and Graves’ disease in Patient 9) and were under rheumatologic follow-up and treatment (Table 2).

### 3.2. Outcome and Follow up Data

All patients had a follow up between 1–18 years (median: 4 years). Contact was lost with Patient 10. Patient 6 was diagnosed with angiopathy after his hospital admission for subdural hematoma 18 years ago (2001), but since then, he was not on any medication or neurological follow up until recently (2018). At the time of the patients’ discharge from the hospital, their disability was moderate as measured by mRS (median mRS: 2.5, range: 0–4). This score remained stable (median mRS: 2.5) despite one definite new stroke in one patient and a probable TIA in another. All of the patients were discharged from our hospital with aspirin, statin, antihypertensive, and antiepileptic drugs in the case of focal seizures. Only one patient underwent neurosurgical intervention at a specialized center abroad.

### 3.3. Neurocognitive Profile

The data on cognitive status were available for 10 patients. One patient was assessed only for his language abilities and one underwent basic cognitive evaluation because of sensory deficit restrictions and demonstrated difficulties in executive and working memory tasks. For eight patients, cognitive assessment revealed that the most common deficits were in the executive and language domains (seven out of eight patients), followed by working memory deficits (five out of eight). The least common problems appeared to be in episodic memory and visuospatial processing. Finally, there was an impact on social cognition.

### 3.4. Radiological Data

**Figure 2:**
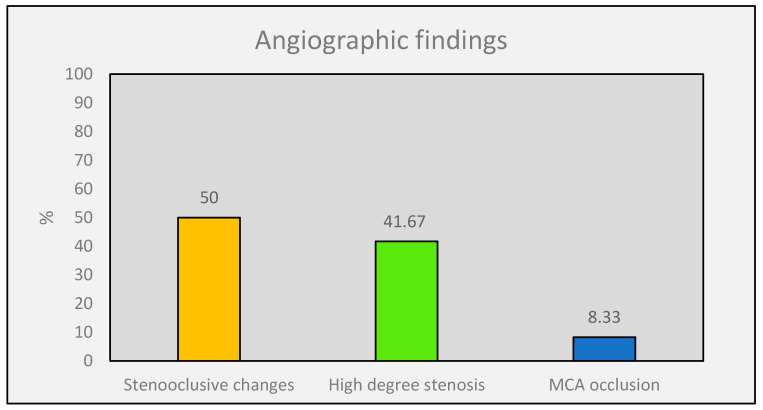
Angiographic characteristics of our Moyamoya population.

In two (16.67%) patients, the MRI did not show any ischemic lesion, neither cortical nor subcortical. Patient 6 had only subcortical chronic ischemic changes at the time of the diagnosis and at the follow-up MRI 18 years later. In Patient 12, the MRI demonstrated deep chronic hemorrhagic changes in bilateral basal ganglia. Three patients had an acute infarct in the superior and one patient in the inferior branch of MCA, whereas in two patients, additional subcortical chronic lesions were also found. Furthermore, four patients suffered from an acute ischemia in the territory of proximal MCA, plus a small cortical branch infarct in one patient and few subcortical chronic lesions in another patient. No patient had an infarct in the ACA or PCA territory (Table 2). All patients underwent MRA and 11 of the 12 patients were examined further by DSA, which confirmed the findings. The median Suzuki score at diagnosis was 4 (25th–75th: 3.5–5, range: 3–5). In our patient population, we did not record any Suzuki score of 1 or 6, nor any other vascular pathology such as aneurysms or AVMs. There was no PCA involvement recorded in any of our patients. The MRA and DSA of patients revealed three main groups of angiographic changes: (1) stenoocclusive changes of one or both ICA, (2) a high degree of ICA stenosis plus stenoocclusive changes of ACA and/or MCA, and (3) complete occlusion of MCA. Stenoocclusive changes in both ICA were identified in six (50%) patients. A high degree of ICA stenosis and stenoocclusive changes in ACA and/or MCA were revealed in five patients (41.67%). One patient (8.3%) had complete occlusion of the left MCA (Figure 2).

### 3.5. Labo Ratory Tests

CSF examination was performed in seven (58.34%) patients (including Patient 3 and 9 with a medical history of an autoimmune disease) and it did not reveal any abnormal findings nor any evidence of CNS involvement due to the autoimmune disease (Table 2).

## 4. Discussion

MMD is a very rare angiopathy outside of the Eastern world; hence, our knowledge is limited. Although most ethnicities have described the disease, the available data are sparse. In Europe, few studies [18,19,20,23,24,25] of Moyamoya adult and children patients have been carried out. In this study, we present the first data of 12 Greek adult Moyamoya patients and their demographic characteristics, symptoms and outcomes, cognitive profiles, and brain images.

In our study, we did not observe the strong female predominance (Female:Male ratio:1.4:1) that has been recorded in other studies and is considered typical for the so-called Western phenotype [9,23,26,27,28,29,30]. Nevertheless, due to the relatively small sample, this result is quite doubtful. Preliminary data from a more recent sample of ours, which are to be published in the immediate future, seem to strengthen the observation of increased frequency in Caucasian women.

The age at disease diagnosis in our population is in line with other studies: older patients (43.5 years) compared with the Eastern data [10,26,31] and slightly older than other Caucasian patients (40.2 years in a Finnish study, 40.5 years in a German study, and 35 years in a more recent German population) [23,24,25]. A possible explanation could be that in some patients, there were preceding non-specific symptoms, such as headaches, short-lasting sensation of tingling, and numbness that were underestimated by the patients or general physicians. A noticeable point is that in our population, there was also a slight difference between sexes and age of disease presentation (female/male: 41 years/44 years), which was not statistically significant. Acker et al. reported a low percentage of family history of the disease [23], but in our patients, we did not record any family occurrence of theangiopathy, in line with previous German and Finnish studies [24,29]. Kraemer et al., however, in 2019, presented familial clustering in 5.7%, implying a more complex genetic background of MMA in Caucasians than what was previously thought [25]. Saarela and colleagues shared the possible explanation of the founder effect, but we also did not record any familial cases even though Greeks are not genetically that isolated as Finnish [28,32]. In our study, only two patients (16.67%) were recorded with a unilateral disease, which was fewer than Finnish patients (23%) [24] and German populations (17.2%, 18.6%, and 23.5%) [16,23,29] and comparable to Japanese data [2,33]. Still, our population was small, thus this finding cannot really lead to any conclusions [28,34].

The common cerebrovascular risk factors such as hypertension, dyslipidemia, smoking, and DM were also confirmed in our patients. The majority had at least one (41.67%) or two (25%), and the most common was hypertension. A possible explanation for this could be the older age of our patients, but it is interesting that in recent publication, hypertension was diagnosed in 50% of patients, even though they were younger [16,25]. In accordance with other studies, almost all of our patients presented with ischemic manifestations (84%), either TIA (16.67%) or TIA, which soon evolved into stroke (41.67%) [35,36]. Only three patients (25%) were admitted to the hospital with stroke without any other preceding symptoms. We have to report a lower recurrence compared with the other European reports [37] based on two patients (Patient 3 with a possible TIA and Patient 9 with a definite new ischemic stroke). Patient 9 had a new small ischemic lesion in the same arterial territory 10 months after the initial diagnosis. Patient 3, one and a half year after first diagnosis, complained of recurrent episodes of numbness in the right side of her body and she was treated with antiepileptic drugs, which led to some improvement. However, as in previous studies, it is difficult to differentiate a focal seizure from a TIA [29].

The hemorrhagic manifestation of the disease in our adult population was lower compared with all Eastern studies [35], although higher than referred for Caucasians (16.67% vs. 7.8 vs. 13%, respectively) [23,24]. Subdural hematoma, which was manifested in one of our patients (Patient 6), has never been referred to as a manifestation; thus, we assume that there was probably another trigger factor (e.g., minor trauma) that led to this brain lesion and that to the incidental diagnosis of vasculopathy.

In accordance with other studies, we recorded other comorbidities and diseases, most common of which were headaches [38], which did not have any specific characteristics (not necessarily migraines).

As far as the stroke severity and disability of our patients is concerned, these were moderate. Few studies have reported patients’ disability [33,39] after diagnosis. The mRS at discharge remained stable in the follow-up visit.

There is limited evidence about the neurocognitive status in the Caucasian population. The high frequency of executive and language deficits in our sample is in accordance with previous studies reporting impairment in these domains in Moyamoya disease, with or without a stroke [40,41]. Surprisingly, in our cohort, cognitive deficits were manifested in almost all patients, which is in contrast with recent publications, where cognitive deficits are reported in only 1.55 of adult patients with MMD [25]. Different methods of cognitive deficit assessments could explain this discrepancy [42].

It is noteworthy that the vascular lesions in Greek patients were restricted to the anterior circulation, which is in contrast with many other studies in Asians and in Caucasians [23]. Most of the Greek patients (50%) had stenoocclusive changes in one or both ICA and only one (8.33%) had a disease restricted to MCA, but none had PCA vascular changes [43]. In Acker’s study, the affected PCA was recorded in a quarter of the patients and this finding is in line with published Asian data [23,44]. In a Finnish population, similar to the German population recently, a significantly lower involvement of PCA (13–16%) was reported [3,24,25], which nevertheless is quite high compared with ours.

Surprisingly, despite the severely affected vasculature, we did not detect any cerebral aneurysms or AVM either at the time of the initial diagnosis or at the follow up [45,46]. This is also a different finding compared with what is recorded not only in Caucasian studies, but also in that of Asian studies [25,47,48].

All patients with ischemia received antiplatelet therapy, statin, and antihypertensives. In our country, surgical treatment for this disease is not available as the disease is rare, the number of patients small, and there is no center specialized in revascularization. Despite the conservative approach [49], the mRS of the patients and their imaging characteristics remained stable over time, a finding that has already been suggested [29].

Our study has specific limitations that need to be addressed. It is a single-center study, with potential referral biases and a small number of patients, even though we did not expect a significant higher number, considering the low incidence of the disease in Europeans [50]. On the other hand, the study has some strengths that have to be underlined. All patients were thoroughly investigated by the same investigators over many years. The study population is genetically homogenous and all patients, except one, had conservative treatment, elucidating the natural history of this rare disease.

Our study adds some new data to the limited information about this angiopathy. The later onset of the disease, ischemic manifestation, and absence of familial occurrence strengthen the postulation of a Western phenotype that is different from the Asian phenotype. Moreover, the absence of any other vascular lesion (AVM) or aneurysms, coupled with the involvement of the posterior circulation over years in our study raise the suspicion for a third phenotype, a Mediterranean one, or at least a probable variation.

In conclusion, there is very little information about Caucasian Moyamoya patients and even less about the Mediterranean population. A European registry is needed to facilitate further research on underlying genetic and racial differences and to confirm the assumption that the disease is a distinct cerebrovascular entity in the non-Asian population.

## Figures and Tables

**Table 1:** Demographic characteristics and presenting symptoms.

Gender	
Female, *n* (%)	7 (58.33)
Male, *n* (%)	5 (41.67)
Female:male	1.4:1
Type of disease, *n* (%)	
Unilateral	2 (16.67%)
Bilateral	10 (83.3%)
Age at first symptom, median (range), years	42.5 (17–49)
Age at diagnosis, median (range), years	43.5 (28–54)
Presenting symptoms, *n* (%)	
TIA	2 (16.67%)
TIA evolving to Stroke	5 (41.67%)
Stroke	3 (25%)
ICB	2 (16.67%)
NIHSS at diagnosis, median (range) *	7.5 (0–23)
Risk factors, *n* (%)	
Hypertension	6 (50%)
Dyslipidemia	5 (41.67%)
Smoking	5 (41.67%)
Diabetes	1 (8.33%)
Number of risk factors, *n* (%)	
0	2 (16.67%)
1	5 (41.67%)
2	3 (25%)
3	2 (16.67%)

*n* = number of patients; TIA = transient ischemic attack; ICB = intracranial bleeding; NIHSS = National Institute of Health Stroke Scale; * NIHSS calculated for 10 patients with ischemic events (TIA/stroke).

**Table 2:** Clinical, radiological, and neurocognitive findings of each patient.

Patient	Age	Type of Disease Unilateral/Bilateral	Presenting Symptoms	Common Vascular Risk Factors Other Associated and Non-Associated Diseases	Outcome mRS (Hospital Discharge→fu) Recurrence	Brain Image (MRI-MRA/DSA)	Cognitive Status
1/F	28	MMD-b	Stroke	No Congenital glaucoma	mRS:4	Acute ischemic lesion in inferior L—MCA + Subcortical chronic small ischemic lesions; High-grade stenosis of both ICA +steno-occlusive changes of ACA-MCA; Collaterals in BG	Impairment in executive functions, working memory. Basic language skills were preserved.
2/F	47	MMD-b	Stroke	Hypertension Congenital cataract	mRS:3→1	Acute ischemic lesion in superior R—MCA; Steno-occlusive changes of both ICA; Leptomeningeal collaterals	Impairment in executive functions, language, episodic memory, working memory
3/F	41	MMD-b	TIA	Hypertension Headache Ankylosing Spondylutis	mRS:2 recurrent TIA (D.D focal seizure)	No ischemic lesions; steno-occlusive changes of both ICA; Collaterals in BG	Impairment in executive functions, language, visuospatial processing
4/M	54	MMD-b	TIA	Dyslipidemia smoking	mRS:0	No ischemic lesions; High-grade stenosis of both ICA +steno-occlusive changes of ACA-MCA; Collaterals in BG/Sylvius	No impairment
5/F	44	MMD-b	TIA→Stroke	Smoking Headache Asthma	mRS:2	Acute ischemic lesion in superior division of L—MCA; Subcortical chronic small ischemic lesions; High-grade stenosis of both ICA +steno-occlusive changes of ACA-MCA; Collaterals in BG	No assessment
6/M	44	MMD-b	Subdural hematoma	No Poliomyelitis	mRS:4	Subcortical chronic small ischemic lesions (2001); Subcortical chronic small ischemic lesions (2019); Steno-occlusive changes of both ICA (2001)/Occlusion of both ICA (2019)	Impairment in executive functions, language, working memory
7/F	49	MMD-u	TIA→Stroke	Hypertension Dyslipidemia Smoking	mRS:2	Acute ischemic lesion in superior L—MCA; High-grade stenosis of L ICA +steno-occlusive changes of ACA-MCA	Impairment in executive functions, language, episodic memory, working memory
8/M	43	MMD-b	TIA→Stroke	Hypertension Dyslipidemia Smoking	mRS:4→3	Acute ischemic lesion in proximal L—MCA; High-grade stenosis of both ICA +steno-occlusive changes of ACA-MCA	Impairment in executive functions, language, visuospatial processing
9/F	40	MMD-b	TIA→Stroke	Dyslipidemia Graves’ disease	mRS:3 recurrent small ischemic stroke	Acute and chronic ischemic lesions in proximal L—MCA; Steno-occlusive changes of both ICA	Impairment in executive functions, language, working memory, visuospatial processing
10/M	47	MMD-u	Stroke	Hypertension Dyslipidemia	mRS:4→lost in fu	Acute ischemic lesion in proximal L—MCA; Occlusion of L MCA	Impairment in language (assessment of the remaining domains unavailable)
11/M	42	MMD-b	TIA→Stroke	Hypertension Smoking	mRS:2→1	Acute ischemic lesion in proximal R—MCA + small L- MCA cortical branch infarction; High-grade stenosis of both ICA +steno-occlusive changes of ACA-MCA	Impairment in executive functions, language
12/F	39	MMD-b	ICH	DM 1	mRS:3	Chronic deep hemorrhagic lesion bilaterally in BG; High-grade stenosis of both ICA +steno-occlusive changes of ACA-MCA; Collaterals in posterior circulation	No assessment

F = female; M = male; MMD = Moyamoya disease; fu = follow-up; ICA = internal carotid artery; MCA = middle cerebral artery; ACA = anterior cerebral artery; BG = basal ganglia; D.D = differential diagnosis; TIA = transient ischemic attack; mRS = modified Rankin scale; DM = diabetes mellitus.

## Data Availability

Data are available from the correspondings author upon reasonable request.
